# Commercial tobacco endgame themes in the Australian media from 2000 to 2021

**DOI:** 10.1136/tc-2023-058186

**Published:** 2023-12-30

**Authors:** Andrew Perusco, Alice Holland, Raglan Maddox, Kylie Morphett, Christina Heris, Coral E Gartner

**Affiliations:** 1 National Centre for Aboriginal and Torres Strait Islander Wellbeing Research, National Centre for Epidemiology and Population Health, Australian National University, Canberra, Australia Capital Territory, Australia; 2 National Health and Medical Research Council Centre of Research Excellence in Achieving the Tobacco Endgame, University of Queensland, Brisbane, Queensland, Australia; 3 The University of Queensland School of Public Health, Herston, Queensland, Australia; 4 UQ Centre for Clinical Research, University of Queensland, Herston, Queensland, Australia; 5 School of Public Health, University of Queensland, Herston, Queensland, Australia

**Keywords:** end game, disparities, media, public policy

## Abstract

**Background:**

Conventional tobacco control is dominated by demand-reduction measures, whereas commercial tobacco endgame (endgame) policies address the key drivers that maintain the tobacco epidemic, such as Tobacco Industry interference in policymaking, the addictiveness of commercial tobacco products and their widespread availability via retail outlets. While Australia has been a pioneer in tobacco control, Australian Governments are yet to commit to endgame policies. The media play an important role reflecting and influencing public opinion and policymaker positions, and can help set the agenda for policy innovation.

**Method:**

Media articles mentioning tobacco endgame goals and policies published between 2000 and 2021 were identified by searching *Factiva* and *Google* (News). We used reflexive thematic analysis (RTA) to identify themes in the articles, supported by content analysis, to describe elements of the data and sentiment analysis to categorise the article sentiment. A deductive–inductive approach was applied in the RTA, coding text from the articles against predefined codes, while also generating new codes where novel themes were identified. Codes were then grouped and summarised.

**Results:**

One hundred and ninety-three articles were included for analysis. The media discourse focused on three policies: tobacco-free generation; banning or phasing out retail supply of tobacco; and mandating a very low nicotine content standard for cigarettes. A broad range of themes in the articles supported endgame policies, including the large health toll from tobacco, government responsibility to act and the total social costs far outweighing any economic benefit from the tobacco market. Opposing themes included the purported failures of ‘prohibition’, illicit trade, ‘nanny statism’ and impact on retail trade. Equity themes were scarce. The benefits of a smoke-free society were described at a societal level, rather than the personal benefits for individuals.

**Conclusion:**

Media articles on the tobacco endgame in Australia generally contained positive sentiment about endgame policies. When engaging with the media, endgame advocates should be aware of, and ready to counter, opposing themes such as the purported failures of ‘prohibition’, ‘nanny state’ rhetoric or a growth in illicit tobacco trade.

**WHAT IS ALREADY KNOWN ON THIS TOPIC**

- The media plays an important role in influencing support and opposition to conventional tobacco control policies.

**WHAT THIS STUDY ADDS**

- This is one of the first papers to explore themes in media reporting on the range of endgame goals and policies. Similar to findings from surveys on public support for phasing out tobacco sales, Australian media articles generally supported adoption of tobacco endgame policies.

**HOW THIS STUDY MIGHT AFFECT RESEARCH, PRACTICE OR POLICY**

- Given the supportive media environment in Australia, this study highlights significant potential for media discourse to advance policies to end the tobacco epidemic, including opportunities for proponents to communicate the potential of endgame policies to improve equity in tobacco-related outcomes. Generally supportive media sentiment should give confidence for governments to enact policy reform to end the epidemic.

## Introduction

 Australia has reduced daily smoking prevalence from 23.8% in 1995 to 10.1% in 2020/2021,^1 2^ but the annual social costs from tobacco use remain high at $A137 billion in 2015/2016,^3^ and are borne disproportionately by some population groups.^4 5^

Tobacco control policy responses have prioritised demand-reduction approaches,^6 7^ including taxation, mass media campaigns, advertising bans, plain packaging and pack health warnings. These policies largely place the onus on individuals to change their behaviour without addressing the key drivers that sustain the tobacco epidemic, such as Tobacco Industry interference in policymaking, the addictiveness of commercial tobacco products and their widespread availability via retail outlets.^6^ While demand-reduction policies denormalise smoking and exert downwards pressure on smoking prevalence, they are associated with stigma for people who smoke^8^ and inequitable outcomes.^9^

Commercial tobacco endgame (endgame) policies focus on the structural determinants of smoking and include: phasing out retail sale of tobacco; banning the sale of tobacco to everyone born after a set year (smoke-free generation/tobacco-free generation (TFG) policies); requiring adults to obtain a consumer licence to purchase tobacco; regulating the contents of tobacco products to reduce their addictiveness by mandating a very low nicotine content (VLNC) standard,^10 11^ and banning features that make smoking more palatable.^12^ Modelling suggests that endgame policies could rapidly and permanently reduce smoking prevalence, while also reducing health disparities.^13^,^15^

Australia is a country with a prevalence target of <5% smoking prevalence by 2030,^16 17^ but has yet to commit to any of the endgame policies described above. However, there has been movement towards endgame policies in the region. For example, the *Tobacco Free Generation* bill was tabled to Tasmania’s Legislative Council in 2014, but was not legislated.^18^ In 2020, Australia’s National Health and Medical Research Council funded the Centre of Research Excellence in Achieving the Tobacco Endgame, to research options for ending the tobacco epidemic in Australia.^19^ Furthermore, Aotearoa/New Zealand (A/NZ), which neighbours Australia, passed laws in 2022 to implement a package of endgame policies to support its goal of <5% daily smoking by 2025 for all population groups, including ending tobacco sales to anyone born after 2008 (TFG), a VLNC standard for smoked tobacco products, and reducing tobacco retail availability by at least 90%.^20^ Australia’s *National Tobacco Strategy 2023–2030* launched in May 2023 makes no firm commitments to implementing innovative endgame policies, but does state that some policies will be explored, including VLNC.^17^ A qualitative study on the views of selected individuals working in tobacco control in Australia suggested conventional policy measures, rather than endgame approaches, remain the focus.^21^ Given that the current rate of decline is insufficient to reach the goal of <5% smoking prevalence,^22^ and current approaches are unlikely to achieve equity for populations with high smoking prevalence, additional support is required to progress policies that could achieve this national goal.

Media coverage of new policy proposals may influence public and policymaker perceptions. Attempts to introduce endgame policies are likely to generate media discourse that supports and opposes such policies.^23 24^ A number of Australian analyses of media articles on tobacco control have been published, including on plain packaging,^25^ electronic cigarettes^26^ and on overall tobacco control discourse.^27^,^29^ No study has explored Australian media discourse on the range of endgame policies. We aimed to understand which endgame policies have been discussed and how the endgame has been portrayed in the Australian media by analysing sentiment towards endgame goals and policies, and supporting and opposing themes/arguments in articles reporting on the endgame.

## Methods

### Reflexivity statement

We recognise that our values, worldviews and biases inform this research and how it was conducted. The research team brings lived Indigenous experience (RM), training in public health (all authors), a range of expertise and various levels of experience in tobacco control (all authors) and in public sector administration (AP, CH, RM, CEG). All authors support the goals of addressing health inequities and reducing smoking to minimal levels to minimise tobacco-related disease and death.

### Search

The *Factiva* database that indexes media articles and the *Google Search* (news) engine were used to identify Australian media articles that mentioned tobacco endgame goals or specific endgame policies as described in a study by McDaniel *et al.*^10^

The search terms used were as follows:

*Factiva*: ‘tobacco free generation’, ‘smoke free generation’, ‘tobacco endgame’; smok* OR tob* AND: ‘filter ban’, OR ‘regulat* palatab*’, OR ‘regulat* flavour’, OR ‘supply ban’, OR ‘avail* ban’, OR ‘disclosure regulat*’, OR ‘nicotin* regulat*’. Articles focused on or about e-cigarettes were excluded by adding NOT vap*, e-cig* to all searches. The ‘NOT’ operator was applied for e-cig* and vap* in the Factiva search, as these terms resulted in too many articles that were irrelevant to that did not discuss endgame goals or policies.

*Google News*: The same search terms were applied, however, the ‘NOT’ operator was not applied, to identify discussing e-cigarettes/vaping as a strategy within the context of endgame goals or policies.

### Inclusion and exclusion criteria

Articles were included if they: (1) were text-based articles (newspapers, online news or major blogs; eg, *The Conversation* or *Croakey*, or transcripts of broadcast media); (2) were published between 1 January 2000 and 31 December 2021; (3) were aimed at the general Australian public; and (4) discussed endgame policies or an endgame goal.^11^ Press releases and trade/industry publications for a technical/commercial audience were not eligible for inclusion. Authors (AP and AH) screened articles independently against the inclusion criteria. Disagreements were resolved with a third author (CG).

### Analysis

Data was analysed in two ways. First, a deductive content analysis and sentiment analysis categorised the overall sentiment toward endgame goals/policies (positive, negative or neutral sentiment).^23^ Articles were classified as supporting or opposing depending on whether the author of the article emphasised supporting or opposing arguments, and/or if a clear position (support or opposition) was expressed. Where there was no clear position for or against the policy expressed, or there was approximately equal balance between supporting or opposing arguments, articles were reported as neutral. Where there was uncertainty, a third reviewer decided the categorisation. A qualitative reflexive thematic analysis, broadly guided by Braun and Clarke’s process,^30^ in NVivo V.12 software (QSR International) was also used to identify, organise and analyse key themes.

Initially, authors AP and AH familiarised themselves with the data by reading all articles prior to coding. AP and AH then used a deductive (predefined codes related to the research questions, and previous literature on tobacco endgame policies and themes) and inductive approach (developing new codes when no suitable code was predefined) to code themes. Predefined codes were used for the following topics and themes: (a) endgame concept/goal and policies^10^; (b) supporting or opposing arguments for endgame policies^23^; (c) equity issues (including related to Indigenous peoples)^31^; and (d) policy image,^32^ (eg, failure/success of tobacco control, world leading, regulatory anomaly or governance themes,^23^ such as ‘social justice’ or ‘market justice’ themes).

Authors AH and AP coded articles across themes a–d. AP coded for theme d. During coding, some nodes were collapsed or expanded and/or the names of the nodes and subnodes were refined to reflect the themes used in the articles. CH double-coded a subset of the articles (10%) for consistency and rigour in an iterative process.

AP then summarised the coded data into dominant (ie, appeared frequently) and minority themes. AP reviewed and refined the themes in an iterative process, including the development of this manuscript. All authors supported refinement throughout this process.

## Results

**Figure 1:**
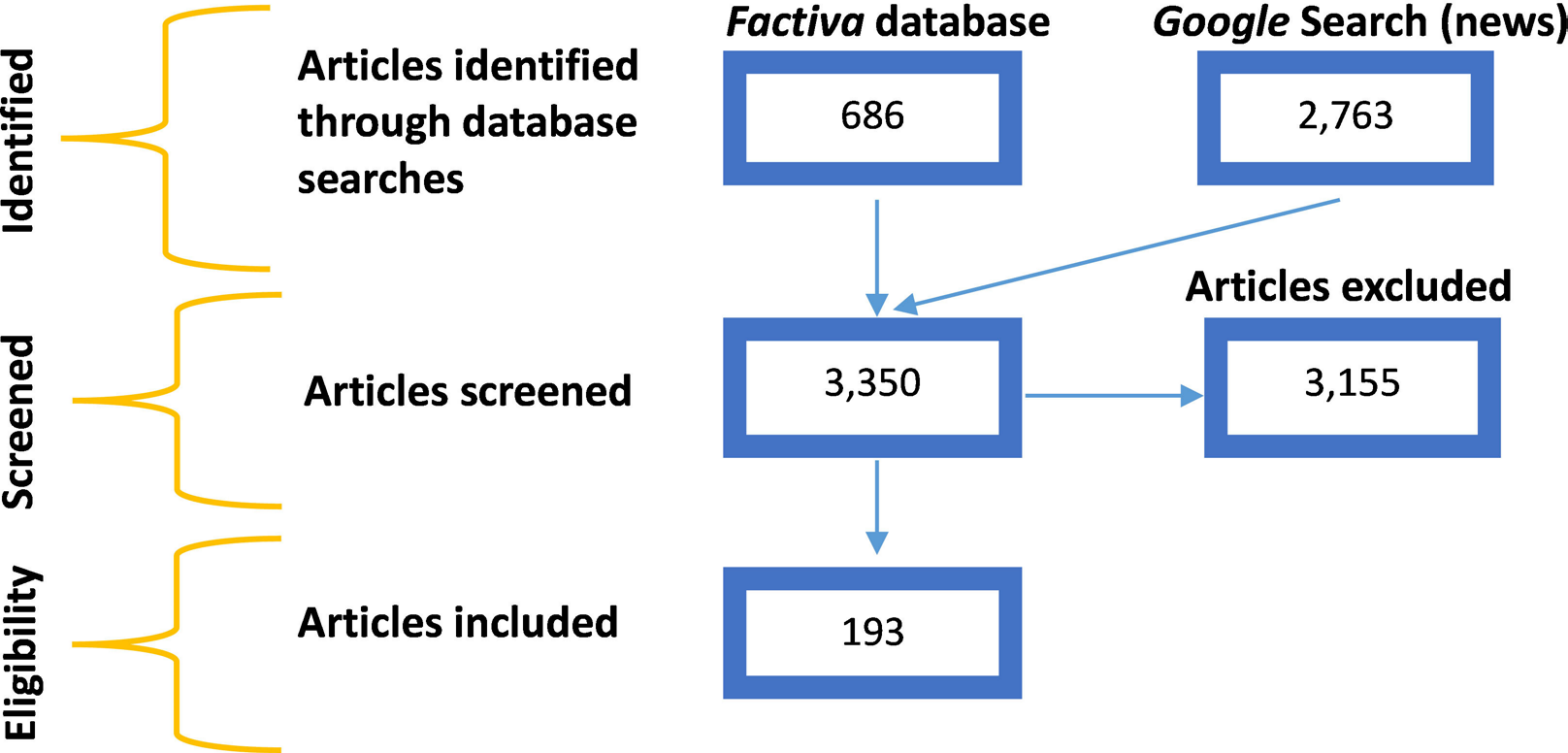
Search results.

We identified 686 articles in the *Factiva* database (figure 1). After removing 99 duplicates, we screened the remaining 587 articles and identified 181 articles that met our inclusion criteria. Of articles identified via the Factiva database search, 356 of the 406 excluded (88%) did not discuss policy within an endgame context, 254 (63%) did not discuss endgame policies, 159 (39%) did not target an Australian audience and 135 (33%) were published in non-news media (eg, trade or professional journals, non-news websites or PR databases). The search via Google Search identified 2763 articles; 13 of these met the inclusion criteria and were not duplicates from the Factiva search results. A total of 193 unique articles met the inclusion criteria.

**Figure 2:**
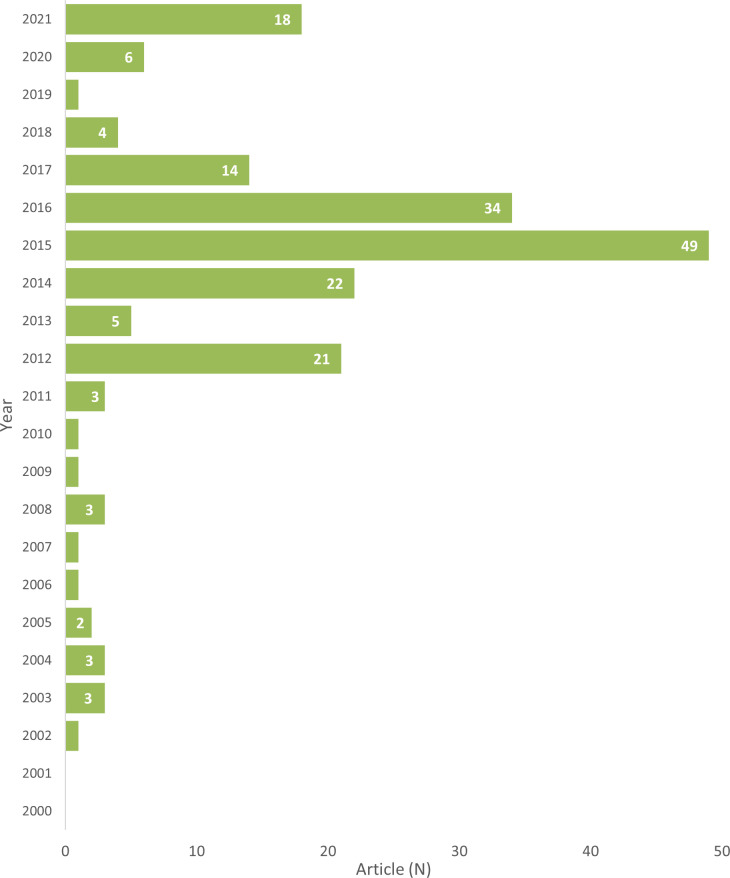
Media articles by year of publication.

Figure 2 depicts the frequency of articles by year of publication, and shows that the number of articles peaked from 2012 to 2016, followed by another peak in 2021. There was a wide range in the number of articles per year with a peak of 49 articles in 2015, only one article identified in the years 2002, 2007, 2009, 2010, 2019 and no articles identified in 2000 or 2001. The peak in the 2012–2016 period relates to debate surrounding draft legislation in Tasmania (ie, the *Tobacco-free Generation Bill 2014*) that would have made it illegal to supply tobacco to everyone born after 2000, from 2018.^33^ The peak of articles in 2021 related to commencement of consultation on the Aotearoa/New Zealand Smokefree Action Plan in 2021. Most articles were news articles (N=127; 66%), and the remaining were commentaries/editorials (N=37, 19%), and letters (N=29, 15%).

**Figure 3:**
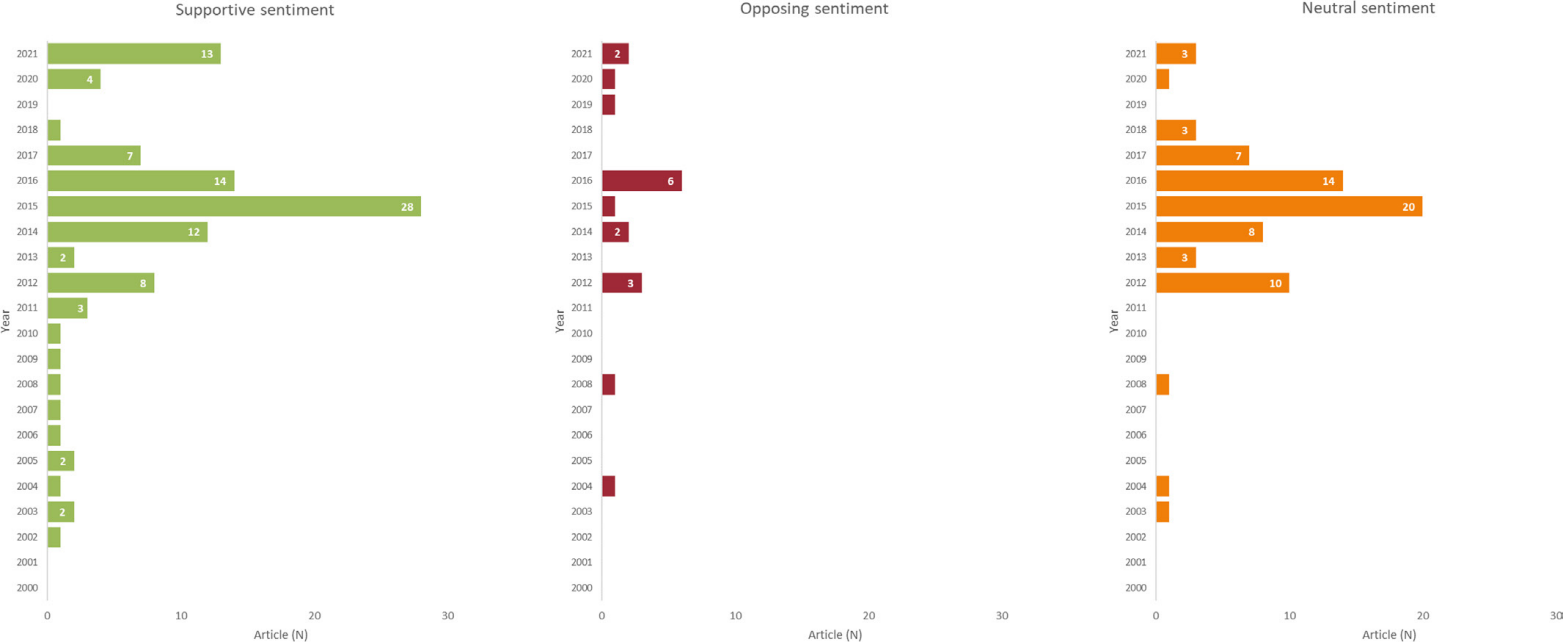
Media article sentiment by year of publication.

Figure 3 depicts sentiment across all the articles by year; overall most were supportive (N=103, 53%), followed by neutral (N=72, 37%) and opposing (N=18, 9%). In 2015 and 2016 (the years in which there was heightened media discourse related to the TFG Bill in Tasmania), 51% of articles supported endgame policies. In comparison, 82% of articles supported endgame policies in 2021 (a year of heightened media discourse while there was no policy under active consideration in Australia). In 2015 and 2016, 33% of news articles were supportive and 79% of editorial articles were supportive, while in 2021, 77% of news articles were supportive, as were 60% of editorial articles and there were no letters identified.

### Themes

#### Endgame goals, targets and time frames

Twenty-nine articles explicitly defined an endgame. Some definitions were related to specific policies, such as ending or phasing out the retail sale of tobacco. Several articles broadly defined an endgame as making a nation ‘smokefree’, or ‘eliminating smoking’. Some articles described targets of less than 10% smoking prevalence, as a path toward eliminating smoking. Some articles (as early as 2003) also mentioned aspirational aims of less than 5% smoking prevalence with uncertain time frames, but in one instance with a target of the year 2050. An article in 2003 noted an aim of 2–3% smoking prevalence, but targets of 5% daily smoking prevalence were common in articles in 2020 when an Australian target of 5% or lower smoking prevalence by 2030 was stated in the *National Preventive Health Strategy*.^16^ This target was also common in articles in 2021 when A/NZ announced consultation on their Smokefree Action plan, with some articles noting that all ethnic and social groups were included in the A/NZ Smokefree target. Discussion about specific Australian targets for population groups with a high prevalence of smoking was absent from the media articles.

#### Endgame policies

**Figure 4:**
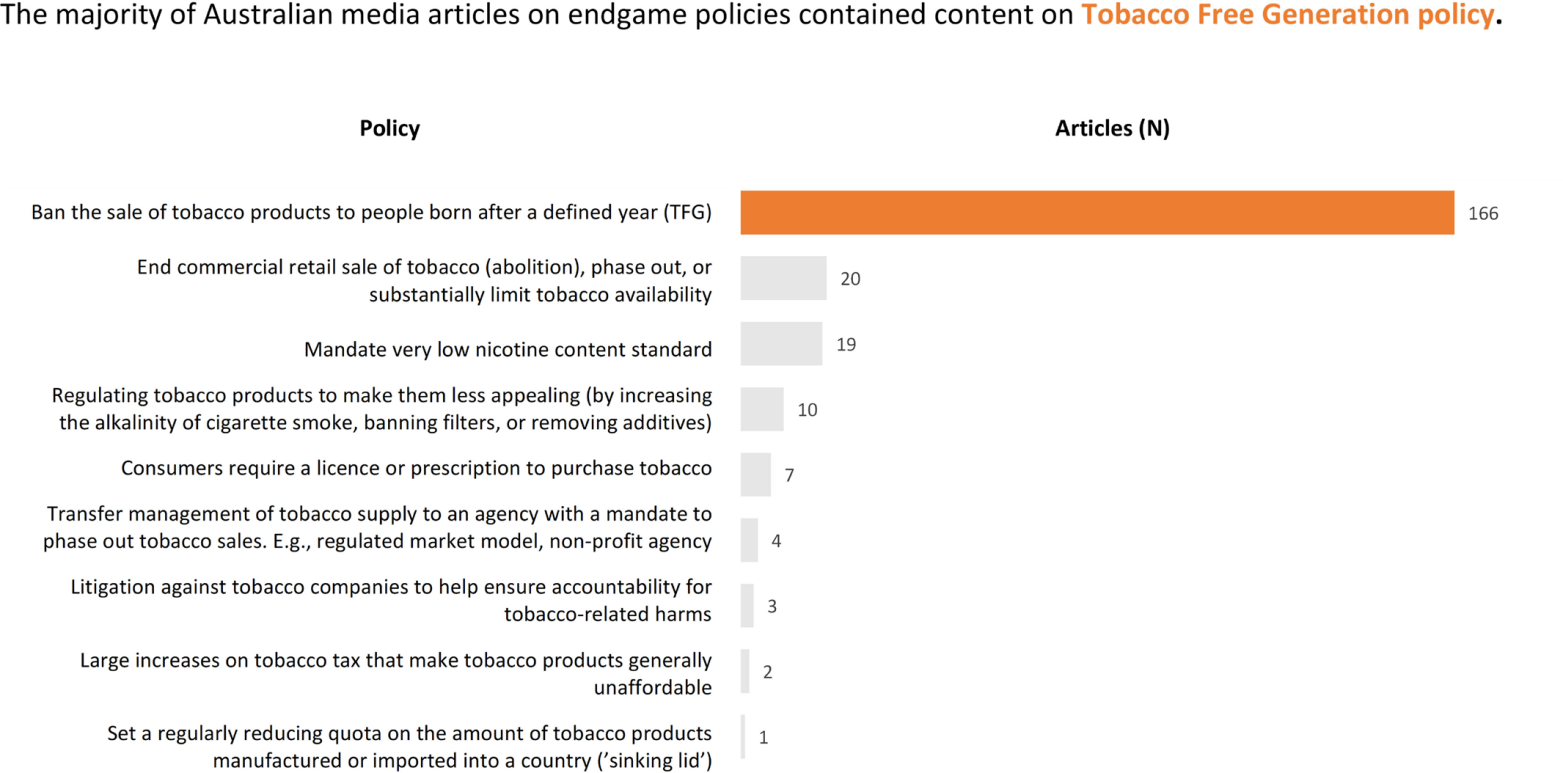
Frequency of media articles mentioning endgame policies. TFG, tobacco-free generation.

The media articles mentioned 9 of the 13 endgame policies categorised by McDaniel *et al*.^10^ Those most often discussed in the policy discourse were: TFG in 166 articles (86%); banning or restricting the retail sale of tobacco products in 20 articles (10%); and mandating a VLNC standard for cigarettes in 19 articles (10%) (see figure 4). Few articles discussed other policies.

##### Supporting and opposing themes

**Figure 5:**
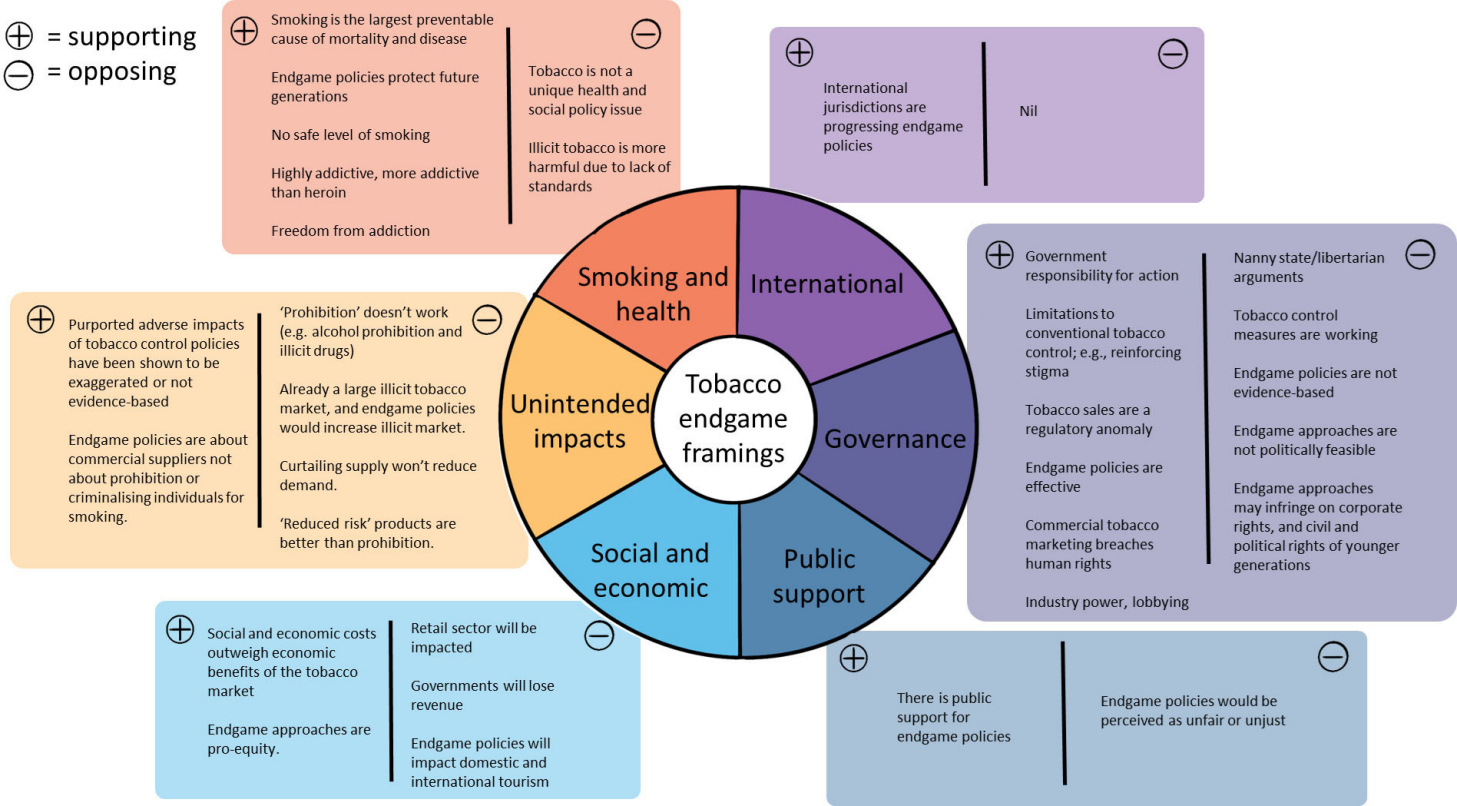
Themes in articles.

Figure 5 summarises the themes identified across the articles, grouped by sentiment. There were a total of 316 items coded as supporting themes across the media articles and 249 items coded as opposing themes.

The large health burden related to tobacco was the dominant supporting theme. Articles that mentioned the disease burden included facts on the size of the problem, and sometimes compared tobacco to other health risks associated with fewer deaths but greater regulatory response. Governments’ responsibility to act was also a dominant supporting theme.

The purported failures of ‘prohibition’ and illicit trade were the dominant opposing themes, followed by ‘nanny state’/libertarian themes. The impact on government revenue was also a commonly cited opposing theme, as was impact on retailers, particularly in the context of attempts to legislate a TFG in Tasmania.

Some articles cited academics who stated that Australia was a policy innovator at the time of plain packaging or stated that Tasmania could be a world leader through implementation of TFG legislation. Some recent articles quoted academics and politicians stating that Australian jurisdictions are now lagging A/NZ. One editorial also suggested Australia should follow A/NZ’s lead. Two articles noted that Australian policy stakeholders would be monitoring the effectiveness of A/NZ’s endgame policies.

Equity themes were rarely seen in the articles. Across four articles, journalists, academics and a politician portrayed A/NZ’s policies as offering significant equity benefits for Māori, Pacific, ethnic and social minority groups, with one of the articles quoting a politician stating that policies will reduce retail outlets in low-income communities. Three articles noted that Aboriginal and Torres Strait Islander peoples are a priority for tobacco control, including one citing the priority within practice guidelines, and another citing an academic. One article quoted an academic who mentioned the importance of including Aboriginal and Torres Strait Islander peoples in decision-making for tobacco control and endgame policies.

### Sentiment among experts and health advocacy organisations

During the mid-2000s, there were few articles overall, but some quoted tobacco control experts who expressed reservations about the value of endgame policies for Australia. However, by 2012 a range of endgame policies were discussed, including an article written by a prominent expert outlining a range of endgame policies, and there was concerted advocacy in the media for a TFG policy in Tasmania between 2012 and 2017. Articles between 2012 and 2015 also reported that health advocacy organisations in Tasmania supported the Tasmanian TFG proposal. However, overall, we found minimal reporting on health organisation positions in relation to endgame policies in Australia. A few articles reported the Australian Council on Smoking and Health advocated phasing out tobacco retail sales.

In 2020 and 2021, several articles noted support for a range of endgame policies by an academic expert, who stated that phasing out retail supply of tobacco was ‘supported by a growing number of tobacco control organisations’. Some articles reported on the creation of a Centre of Research Excellence on the Tobacco Endgame funded by the National Health and Medical Research Council and noted they were exploring and advocating for a range of endgame policies.

## Discussion

### Key findings

Most media articles contained arguments supportive of endgame policies. The media discourse in Australia focused predominantly on TFG, related to the unsuccessful Tasmanian *Tobacco Free Generation* bill.

A broad range of themes in the articles supported endgame policies, such as the large health toll from tobacco, government responsibility to act and the total social costs far outweighing any economic benefit from the tobacco market. Opposing themes included the purported failures of ‘prohibition’, ‘nanny statism’ and impact on retail trade. Equity themes were scarce. Articles in 2021 noted Australia was a tobacco control laggard at commencement of consultation on the A/NZ Smokefree Action Plan.

### Sentiment

The supportive sentiment of most articles was notable considering that approximately 70% of Australian newspapers are owned by one company,^34^ known for propagating ‘nanny state’ rhetoric,^35^ and that significant campaigns against the implementation of tobacco plain packaging legislation were waged in the Australian media by the Tobacco Industry and retailer associations.^36 37^ Australia has been a substantial contributor to endgame discourse in the academic literature, and Tasmania was the first jurisdiction to attempt to implement a TFG law.^7^ Some evidence indicates that news articles were less likely to have a supportive sentiment when a policy is considered for legislation; this is consistent with research showing that proposed tobacco legislation can be hotly contested when actively considered by governments.^36 37^

### Endgame policies

Policies that have progressed along the policy pathway (ie, a bill submitted to parliament) received the greatest focus in the media (N=166, 86% of the articles included in this study mentioned TFG). Australian media articles reported on policy innovations adopted internationally, in particular A/NZ and the Netherlands. Policy research indicates that international experience of policy change has been an important influence on tobacco control policy adoption in Australia,^38^ and provides important opportunities to garner further domestic support in the media.^7^ While the UK government’s adoption of a TFG policy is encouraging,^39^ it does raise concern that adoption of TFG becomes the only endgame policy of choice among countries in late stages of the tobacco epidemic. TFG policies have potential to prevent whole generations of young people from commencing smoking and to reduce tobacco-related inequities among next generations, but modelling suggests they will not lead to rapid changes in smoking prevalence as they do not cater to existing cohorts of people who smoke.^15^ Consequently, the challenge remains for endgame proponents to use the media to promote the benefits of policies such as mandating VLNC, or substantially reducing retail density, which will deliver benefits for everyone. Commitments to *Closing the Gap* in health and life outcomes for Aboriginal and Torres Strait Islander peoples provides leverage for the adoption of policies that will lead to more rapid progress in ending the commercial tobacco epidemic in Australia.^40^

While the recent reversal of endgame policy commitments in A/NZ may dampen prospects for the adoption of endgame policies in other nations,^41^ it provides additional opportunity for endgame proponents to communicate the rationale for endgame policies, and to encourage other countries to lead these policy innovations.

### Supporting and opposing themes

Greater emphasis on the role of environmental/commercial factors as drivers of tobacco addiction can assist in: focusing the lens on the Tobacco Industry’s role in making tobacco products omnipresent; and countering arguments that illicit trade would flourish with the implementation of endgame policies. Endgame proponents should consider directly challenging the validity of ‘prohibition’ themes. These themes generally fail to acknowledge the comparatively low use of illicit drugs (eg, cannabis is the most frequently used illicit drug in Australia, yet only 1.7% of Australians used cannabis recreationally on a daily basis in 2019).^42^ Media discourse could also explore why alcohol prohibition themes are used to oppose policies, when countries such as Taiwan (at the end of WWII) used opium endgame policies to eliminate opium smoking.^43^ Those advocating for endgame policies should emphasise commercial actors/commercial supply, and that endgame policies aim to end the harms caused by commercial tobacco use, not to criminalise or penalise personal use, purchase, or possession.

The dearth of equity themes is concerning given that endgame policies, including TFG, show great promise for public health equity.^15 44^ While some have argued that equity themes may strengthen support only among those who hold similar values,^23^ equity for Māori was a key rationale in calling for and adopting endgame policies in A/NZ.^20^ Unlike Australia, A/NZ set a target of ≤5% smoking prevalence by 2025 for Māori and non-Māori.

Media campaigns focused on industry denormalisation can reduce smoking prevalence,^45 46^ yet few Australian mass media campaigns have focused on the role of the Tobacco Industry in the epidemic.^47^ As there have been recent commitments to national tobacco media campaigns in Australia,^46^ endgame proponents should consider supporting campaigns which denormalise the Tobacco Industry, to encourage the discourse to focus on Tobacco Industry culpability, and strengthen existing public support for endgame policies.

Several articles focused on the potential impact on the retail sector in relation to proposed regulation, that is, Tasmania’s *Tobacco Free Generation* bill. Themes about impacts on the retail sector will likely continue to be a dominant opposing theme considering that they have been used to oppose NZ’s endgame policies,^48 49^ and other tobacco regulation.^36 37^

The benefits of a smoke-free society were described at the societal level, and in economic or epidemiological terms, such as ‘largest cause of preventable mortality or disease, or ‘social and economic costs’. However, guidelines for health communications note that messaging about personal benefits can be more persuasive.^50^ Themes about the personal benefits of smoking cessation (eg, reducing stress and common mental health symptoms)^51^ provide an opportunity to promote endgame policies as a support to smoking cessation, which could further enhance consumer support for endgame policies.

The image of Australia as world leading in tobacco control was justifiable during implementation of plain packaging, expanded graphic health warnings on packaging and commencement of the Tackling Indigenous Smoking programme.^52^ However, themes in some more recent media articles challenged this portrayal, particularly in the context of the development of a smokefree action plan in A/NZ that included endgame policies. Australia is currently implementing strong policy on vaping products and a range of new tobacco control measures that continue an incremental, demand-reduction approach to reducing smoking. It is unclear what impact a recent change in tobacco control policy in A/NZ following a change in government in 2023 will have on policy in other countries, including Australia. Repeal of A/NZ’s world-leading smokefree laws could delay implementation of these policies in Australia or they could provide an opportunity for Australia to show the strong leadership on ending the tobacco epidemic that now appears to be lacking in A/NZ.

### Strengths and limitations

Broadcast media were excluded from this study unless the respective transcripts were available in the databases (n=3). However, newspaper and online news articles provided most of the news stories over the news cycle in Australia.^34^ Consequently, this study provided a comprehensive representation of the range of themes in the endgame discourse in the Australian media.

A small number of the included endgame articles (N=9) discussed ‘reduced risk’ products and some themes were included in this analysis. More focused research on the themes related to e-cigarettes in media reporting is warranted, both in countries such as A/NZ,^53^ England,^54^ USA^55^ and Canada,^56^ which consider access to e-cigarettes as a policy that makes the introduction of greater restrictions on tobacco products more feasible, and in countries that have rejected e-cigarettes as a contributor to a commercial tobacco endgame, such as Finland and Australia.^46 57^

### Conclusion

Australian media reporting of endgame goals and policy options over the last two decades largely centred on the unsuccessful *Tobacco Free Generation* bill in Tasmania, and most recently on the Smokefree Aotearoa 2025 Action Plan, and the creation of an Australian Centre of Research Excellence on the Tobacco Endgame. The tone of media articles was generally positive, suggesting a receptive and supportive media environment for advancing a commercial tobacco endgame in Australia. As media articles tended to centre on key events, endgame proponents can anticipate future opportunities to disseminate supportive themes in the media and to counter opposing themes, and attempt to broaden the discourse beyond TFG policies.

## Data Availability

Data are available upon reasonable request.
